# Preparation Process of In Situ MgB_2_ Material with Ex Situ MgB_2_ Barrier to Obtain Long Sections of Superconducting Multicore Wires

**DOI:** 10.3390/ma18010126

**Published:** 2024-12-31

**Authors:** Krzysztof Filar, Artur Kawecki, Andrzej Jacek Morawski, Eliza Sieja-Smaga, Tomasz Cetner, Andrzej Mamala, Jacek Skiba, Grzegorz Gajda

**Affiliations:** 1 Institute of High Pressure Physics PAS, Sokolowska 29/37, 01-142 Warsaw, Poland; am@unipress.waw.pl (A.J.M.); tcetner@unipress.waw.pl (T.C.); skiba@unipress.waw.pl (J.S.); 2 Faculty of Non-Ferrous Metals, AGH University of Krakow, Mickiewicza Av. 30, 30-059 Krakow, Poland; esmaga@agh.edu.pl (E.S.-S.); amamala@agh.edu.pl (A.M.); 3 Division of Agrotechnological Systems Engineering and Work Safety, Institute of Agricultural Engineering, Wrocław University of Environmental and Life Sciences, 25 Norwida St., 50-375 Wroclaw, Poland; grzegorz.gajda@upwr.edu.pl

**Keywords:** ex situ MgB_2_ barrier, MgB_2_ wires, Mg diffusion, Cu shield, critical current density, long multi-core superconducting wires, Cu-Ag rod, Cu-Ag wires, MgB_2_ powder, hydro-extrusion

## Abstract

In the present study, our emphasis was directed towards the fabrication process of long multi-core superconducting wires, each spanning several hundred meters. These wires feature an in situ MgB_2_ core, an ex situ MgB_2_ barrier, and a copper shield. The cost-effectiveness of these constituent materials, coupled with a judicious arrangement of internal components, facilitates the utilization of an economical shielding material for the resulting wire. Our ongoing efforts have successfully yielded several hundred-meter-long wire sections possessing favorable superconducting characteristics, making them suitable for self-field applications, such as direct current (DC) power lines.

## 1. Introduction

To approach the utilization of MgB_2_ material in industrial or scientific applications, several enhancements are necessary. One of the most important ways to improve the mechanical properties and critical parameters of superconductivity is to incorporate various dopants into the starting material [1,2,3,4,5,6,7,8,9,10]. Another strategy is to investigate a variety of wire core sheath materials [11,12,13,14,15,16,17,18,19], as well as to explore different heating processes [1,20,21,22]. Additionally, a barrier has been considered to separate the core from the sheath to counteract potential reactions [22,23,24,25,26]. Previous studies have shown that copper, used as a shielding material, reacts with in situ MgB_2_ material [15,27]. In such cases, copper penetrates MgB_2_, exhibiting significant diffusion when in contact with in situ MgB_2_. To avoid this, the more expensive niobium, which does not react with in situ MgB_2_, has been used [28]. However, studies indicate that cooper penetration is negligible in ex situ MgB_2_ (pre-reacted material). Therefore, an ex situ MgB_2_ barrier has been proposed, enabling the use of the more affordable copper shielding [29].

This paper focuses on the preparation of a superconducting material using the latter approach, aiming to achieve extended lengths of multi-core wire. The method employed involved backfilling previously prepared capsules, which were then subjected to the hydro-extrusion process [30,31,32,33,34]. Subsequently, the wire drawing process was applied, compacting the obtained single-core wire sections into a single sheath, followed by further drawing to form multi-core wires. Finally, a heat treatment process was carried out. This paper presents experimental results from various stages of the multi-core wire manufacturing process and provides examples of their fundamental critical parameters.

## 2. Materials and Methods

The process for manufacturing MgB_2_ multicore superconductor wires involves several key steps. First, magnesium (Mg) and boron (B) powders are thoroughly mixed to ensure a uniform starting material. The mixed powders are then encapsulated into cartridges, followed by the application of pressure to compact the material. Simultaneously, CuAg0.1 billets are continuously cast and subsequently machined to create capsules designed to house the superconductor materials. The capsules are filled with the Mg and B powder cartridges, along with ex situ MgB_2_ powder, forming the precursor material. Hydro-extrusion is then applied to the filled capsules to form rods, which are processed further into single-core superconductor rods. An optional heat treatment step can be performed at this stage to enhance the material properties. The single-core wires are then compacted into an outer tube layer, forming a multicore rod structure. This multicore rod is drawn through a series of dies to achieve the desired dimensions, resulting in multi-core wires. Finally, heat treatment is performed to synthesize in situ MgB_2_, completing the manufacturing process.

The in situ MgB_2_ material was composed of pure metal powders. The first precursor utilized was magnesium powder with a purity of 99.8% and a grain size of <44 µm, sourced from abcr GmbH [35]. The second precursor was an amorphous boron powder with a purity of 99.1% and a particle size of <0.5 µm, obtained from Pavezyum Kimya [36]. Ex situ MgB_2_ was procured from Pavezyum Kimya, possessing a material purity exceeding 95.0% and a particle size of <35 µm. The outer sheath of the single-core wires was fabricated from a CuAg0.1 alloy. Alloys with low silver content, such as CuAg0.1, find applications in the electrotechnical and energy industries due to their enhanced mechanical properties, increased heat resistance, and high electrical conductivity. Rods of CuAg0.1 alloy with a diameter of 26 mm were produced using a laboratory installation for continuous melting and horizontal casting. Materials in the form of silver granulate with a high chemical purity of 99.99% and oxygen-free copper (Cu-OF) were melted at 1180–1190 °C in a graphite crucible placed within a medium-frequency induction furnace (Thermetal, Dudley, UK). These materials were cast into rods. Graphite granulate was used as a deoxidizer for the liquid metal, while nitrogen provided a protective atmosphere within the crucible. A high-purity graphite continuous casting mold (grade 4450) and a copper primary cooler were employed, along with direct water spray for secondary cooling of the solidified material after exiting the mold. Table 1 provides the parameters of the rod manufacturing process. The strength and electrical properties of the CuAg0.1 alloy rods are presented in Table 2. Uniaxial tensile tests were performed on a Zwick/Roell Z020 testing machine [37]. The gauge length was 200 mm, and the testing velocity was 50 mm/min. The rod manufacturing processes were conducted at the AGH University of Krakow. The CuAg0.1 alloy, based on oxygen-free copper, exhibits very high electrical conductivity, rated at 100% on the IACS scale (International Annealed Copper Standard: 58 MS/m = 100% IACS).

**Figure 1:**
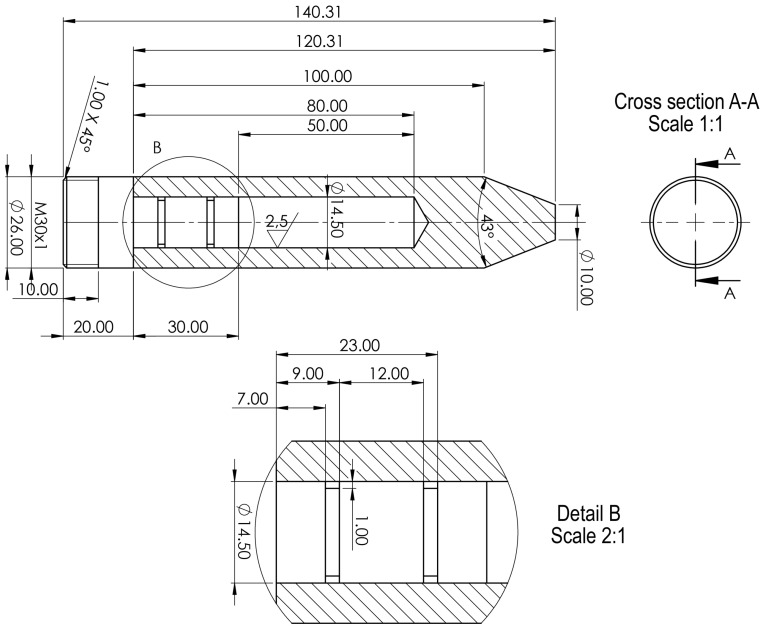
Capsule diagram for hydrostatic extrusion process of the tested materials.

In rods with a diameter of 26 mm made of CuAg0.1 alloy, a hole with a diameter of 14.5 mm was drilled. Within this cavity, a two-layer composition of powders was carefully placed, forming the core (Figure 1). Before starting the drawing process, rod made of CuAg0.1 was subjected to heat treatment at 350 °C for 1 h, which was assumed to eliminate internal stresses in the matrix material.

**Figure 2:**
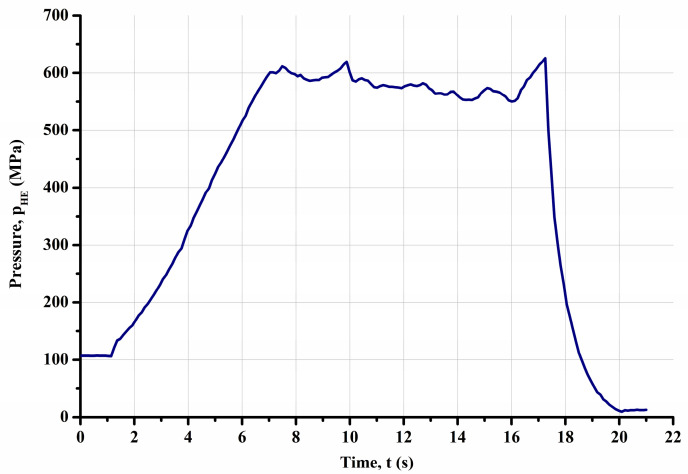
Pressure characteristic of the cold cumulative hydrostatic extrusion of sample.

To prepare the insert for the hydro-extrusion process, a two-stage method was employed. Pre-weighed portions of in situ MgB_2_ powder were carefully introduced into polyurethane tubes with an internal diameter of 8 mm and a length of 110 mm within a glovebox with an argon gas atmosphere (high purity 5N). Subsequently, the cartridges prepared in this manner were pressurized (150 MPa) using a hydraulic press from PPHU POWŁOKA S.C. (Karczew, Poland). The resulting in situ material was centrally positioned within 14.5 mm diameter holes in previously prepared capsules and then covered with ex situ MgB_2_ powder to fill the voids. The capsules, now supplemented in this way, were sealed with a copper stopper. Subsequently, the prepared material underwent the hydro-extrusion process (HE). Hydro-extrusion, a method falling under the category of severe plastic deformation (SPD) techniques, ensures a high level of structural homogeneity in deformed materials while maintaining remarkable process efficiency. This technology has been extensively developed over many years at the Institute of High-Pressure Physics of the Polish Academy of Sciences, primarily utilized for analyzing material susceptibility to high plastic deformation. Years of research conducted under high hydrostatic pressures have affirmed the unique capabilities of the HE method in imparting to deformed materials properties that are unattainable through conventional manufacturing methods such as extrusion, drawing, or rolling. The microstructure fragmentation achieved in tested materials has led to the emergence of entirely new mechanical [38,39], physical [40,41], and functional [42,43] properties. The hydrostatic extrusion process also facilitates the consolidation and extrusion of powder materials [44,45]. Unlike conventional extrusion, where a piston presses against the material, hydrostatic extrusion involves forcing the material through a matrix using a medium compressed to high pressure. The pressure medium envelops and applies hydrostatic pressure to the entire material undergoing deformation, including the deformation zone within the matrix. This significantly impedes the generation and propagation of cracks, extending the deformation process without causing undue cracking in the processed material. These processes were conducted at the Institute of High Pressure Physics of the Polish Academy of Sciences. The hydro-extrusion (HE) process took place on presses specifically designed and manufactured at IHPP PAS Unipress. These presses can operate at pressures up to 1800 MPa and are equipped with a system for cooling the extruded product with cold running water to minimize the effect of adiabatic heating [46,47]. The HE process was performed using a low-angle shaping die with an apex angle of *α* = 45°, at a processing speed of 6 mm/s. A high-pressure medium consisting of a mixture of 10% methyl alcohol and 90% oil by volume was utilized. Stable “flattening” extrusion characteristics were observed at each hydroextrusion stage. The extrusion pressures for the A_Mono and B_Mono samples were recorded at 576 MPa (Figure 2). The pressure value was calculated as the arithmetic mean of 80 measurement points taken from the interval corresponding to the hydrostatic extrusion process, as illustrated in Figure 2. The standard deviation of the measurement series was employed to quantify the measurement error. This approach is valid under the condition that the minimum number of measurements, *n*, exceeds four. In the present study, *n* = 80 measurements were used. The observed slight pressure fluctuations result from the compaction of the powder material during deformation, while the peak increase in the initial pressure in the hydro-extrusion process is related to exceeding the yield point of the solid copper capsule material and overcoming static friction in the tribological system. The single-core material product obtained in the hydro-extrusion process had length of 875 mm for both samples (A_Mono and B_Mono). The hydro-extrusion process parameters are detailed in Table 3.

The bars obtained from the extrusion process were designated for wire drawing in two technological variants and stages: single-core wire drawing and multicore wire drawing. In the first variant (sample A_Multi), wires were drawn to their final diameter without any inter-operational heat treatment. In the second variant (sample B_Multi), heat treatment at 350 °C for 1 h was applied before the drawing process to relieve internal stresses in the matrix material and enhance the plasticity of the coating material. The subsequent process of creating extended lengths of superconducting wire took place at AGH University of Krakow. The material was drawn with a similar elongation coefficient (λ ≈ 1.12) (Table 4) in all wire drawing operations, where the coefficient of elongation is the ratio of the material length before and after the plastic deformation operation, mathematically equal to the reduction ratio (*R*). Table 4 summarizes the diameters to which the tested materials were drawn. The wires were reduced from an initial diameter of 12.15 mm to a final diameter of 1.17 mm.

The cold deformation process was carried out using a laboratory draw bench for drawing rods and wires. The setup comprised a bench drawing machine (length: 5 m; force: 40 kN, chain drive), a measuring system equipped with a Hottinger Baldwin Messtechnik membrane force sensor, a Spider 8 signal amplifier from Hottinger Baldwin Messtechnik, and a portable computer for recording drawing forces during deformation. Conical dies manufactured by Technodiament with a 14 degrees cone angle were employed, incorporating inserts made of sintered carbides (12.15 mm to 3.14 mm) and polycrystalline diamond (3.0 mm to 1.17 mm). Parameters of the wire drawing process, including total deformation, drawing angle, drawing speed, and lubrication, are outlined in Table 5 and Table 6.

Final wire heat treatment parameters for synthesizing in situ MgB_2_ material are provided in Table 7.

Following the wire drawing process, the obtained long-length wires underwent the determination of critical temperature (*T_c_*) and critical current density (*J_c_*) through four-probe transport measurements. For *T_c_* measurement, contacts were made by soldering thin copper wires (50 µm) to the wire sheath. The distance between each pair of contacts was approximately 10 mm. The samples were then glued to a copper sample holder using GE varnish with cigarette paper insulation. For *I_c_* measurement, the samples were soldered to copper current leads using an In52/Sn48 soldering iron (Ancaster, ON L9G 4V5, Kanada). The voltage contacts are made of insulated copper wires, connected to the wire shield by standard soldering. The MgB_2_ material was not exposed during electrical measurements and all soldering was performed on the wire’s sheath, made of copper. The samples were cooled within a vacuum cryostat equipped with a Gifford–McMahon cryocooler (Jiangning District, Nanjing, Jiangsu Province, China), covering a temperature range of 7–300 K. Wire resistance during *T_c_* measurement was measured in Delta mode using a Keithley 6221/2182A (Beaverton, OR, USA) setup. For the initial estimation of the critical current, three different current values (2 mA, 20 mA, and 100 mA) were applied using a Zhaoxin KKD-30100D 100A DC power source (Longxi Community, Longgang Subdistrict, Longgang District, Shenzhen, China) and a Keithley 181 nanovoltmeter (Beaverton, OR, USA), within the temperature range of 12 K–40 K in the self-field. The critical current (*I_c_*) value was determined using a 1 μV/cm criterion. Due to the challenge of distinguishing between in situ and ex situ material after heat treatment, the critical current density *J_c_* was calculated for the entire area of MgB_2_. To evaluate *J_c_* solely for the in situ part, which constitutes the main conductive material, the *J_c_* for the entire MgB_2_ material should be multiplied by a factor of approximately 3. The engineering current density *J_E_* was calculated for the entire surface of the wire, encompassing MgB_2_ and shields. Subsequently, after *T_c_* and *J_c_* measurements of short cable lengths, the material underwent microscopic analysis utilizing the Zeiss Ultra Plus scanning electron microscope (Oberkochen, Germany).

## 3. Results

### 3.1. Results of Wire Drawing Process

The wire drawing process was conducted at a relatively low speed under laboratory conditions. The temperatures of all materials measured after leaving the die did not exceed 80 °C.

**Figure 3:**
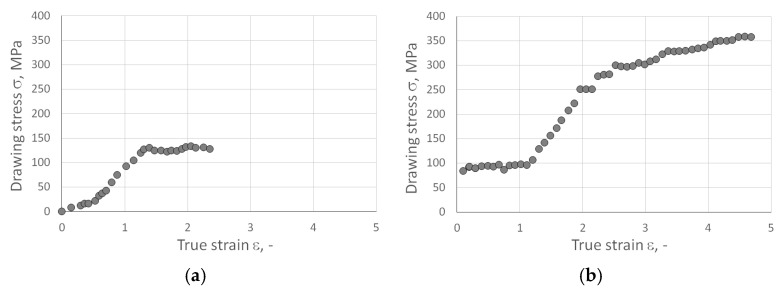
Drawing stress of (**a**) single-core A_Mono wires with a diameter of 12.15 mm to a diameter of 4.15 mm—raw material for A_Multi wire and (**b**) multi-core wires with a diameter of 15 to 1.17 mm as a function of actual deformation.

During the wire drawing process, the drawing force of the wires was systematically recorded. This allowed for the estimation of the drawing stress of the powder-cored wires as a function of their total deformation (true strain). Figure 3 illustrates the values of the average drawing stress for both single-core wires (Figure 3a) and multi-core wires (Figure 3b). The true strain was calculated using the following equation:

(1) ε=ln⁡λ,

where *λ* is the elongation factor and is calculated as follows:

(2) $$\lambda =\ln{\left(\frac{D_{0}}{D_{1}}\right)}^{2}$$

where *D*_0_ is the initial wire diameter (mm) and *D*_1_ is wire diameter after drawing (mm).

**Figure 4:**
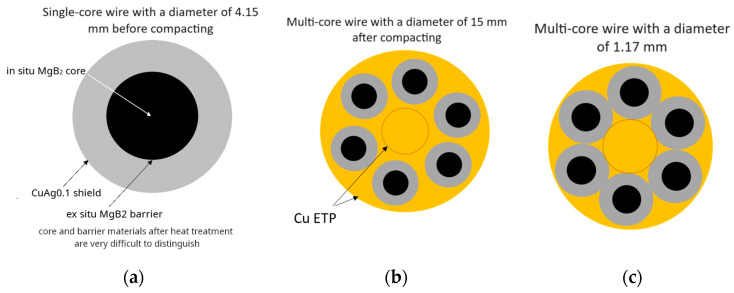
Wires—various stages of manufacturing: (**a**) single-core wires: subjected to a drawing process, reducing the diameter from 12.15 mm to 4.15 mm; (**b**,**c**) multi-core wires: formed by compacting single-core wires with a diameter of 4.15 mm and then subjected to a drawing process, reducing the diameter from 15 mm to 1.17 mm, depending on the actual deformation.

The single-core wires were initially processed to a diameter of 4.15 mm. Subsequently, they were compacted into a 6+1 multi-core system, wherein six wires had a powder core, and one central wire was made of electrolytic copper in the ETP grade. The stages of obtaining multi-core wires are depicted in Figure 4.

During the conventional process of drawing wire from a monolithic material, several factors influence the drawing stress. First, the amount of deformation during the process directly impacts the drawing stress. Second, the material’s average flow stress, determined by factors such as temperature, strain, and strain rate, reflects its resistance to plastic deformation. Third, friction, influenced by the lubricant type, temperature, and drawing speed, significantly affects the wire’s ability to slide through the die. Finally, redundant work, associated with the geometry of the plastic deformation zone, represents work exceeding that required for deformation. The strain in each wire drawing operation is typically limited based on the increase in wire drawing stress. The maximum level of wire drawing stress should be carefully controlled to ensure successful wire drawing. It should be less than the ultimate tensile strength of the drawn wire to prevent breakage during the process. In practice, it is often kept below the yield stress of the drawn wire to avoid permanent deformation of the wire, fulfilling tolerances and surface quality requirements.

In non-conventional wire drawing processes involving a wire with a powder core, special conditions are required. The wire drawing stress is the macroscopic response of the material to plastic deformation. After the wire drawing operation, both the core and sheath experience the same elastic strain in the axial wire direction due to the drawing stress. However, the drawability of the sheath and core can vary significantly. Copper and CuAg0.1 alloy exhibit excellent plasticity, while ex situ MgB_2_ is a brittle material, and the mix of Mg and B powders has low deformability. As a result, the maximum drawing stress is limited by the properties of the core.

The single-core wire drawing stress as a function of total true strain (refer to Figure 3) exhibits three distinct stages. The first stage involves a quasi-linear increase in drawing stress with the increase in total true strain, extending up to approximately 0.5 true strain. The second stage consists quasi-linear increase in the drawing stress with a higher slope, occurring from around 0.5 to about 1.3 true strain. This stage suggests a more pronounced strengthening of the materials. Further research is needed for a clear explanation of this stage. The third stage shows that at true strains above 1.3, a plateau with fluctuations occurs. This stage indicates that the mechanical properties of the wire materials remain largely unchanged despite further plastic deformation. Multicore wire drawing stresses gives more complex behavior. During the process, not only the reduction of diameters of components but also the elimination of empty areas between the components and the change of shape of the components affect the wire drawing stresses.

**Figure 5:**
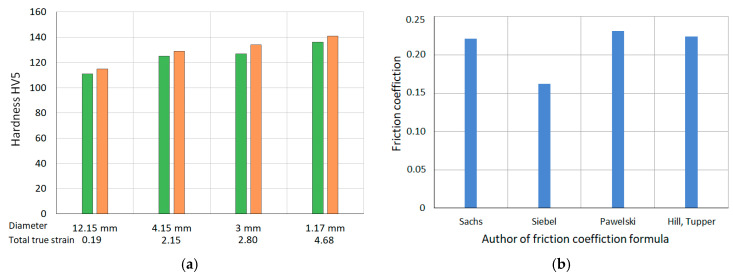
(**a**) Vickers HV5 hardness as a function of changing the diameter of multi-core wires (green color—A_Multi; orange color—B_Multi). (**b**) Average coefficient of friction in the process of drawing multi-core wires A_Multi and B_Multi.

The influence of the extent of plastic deformation on the change in hardness of coating materials, namely, CuAg0.1 and ETP, was investigated. The characteristics of the impact of the amount of plastic deformation on the change in the hardness of coating materials are depicted in Figure 5. Vickers hardness tests were conducted using a Tukon 2500 Knoop/Vickers automated hardness tester (Norwood, MA, USA) with a motorized table, manufactured by Wolpert–Wilson. The test load was 5 kgf (accuracy: ±1% of the load measurement value; ±5 µm for camera distance measurement), and the indentation time was 10 s, with a magnification of 10 times. Table 8 presents the average hardness values of the tested materials. The Vickers hardness of the analyzed materials is approximately 60 HV in the soft-annealed condition. The results show values of 108–136 HV for CuETP and 111–141 HV for CuAg0.1, indicating very similar hardness levels for both materials. Utilizing the well-known Tabor formula, it is possible to estimate the yield stress of metallic materials based on hardness. For the analyzed materials CuETP and CuAg0.1, yield stresses in the range of 380–500 MPa correspond to hardness values of 110–140 HV. This implies that wire drawing stresses (see Figure 3b) increase from 0.3 to 0.7 times the yield stress of the coating and sheath with an increase in total true strain during multicore wire drawing.

**Figure 6:**
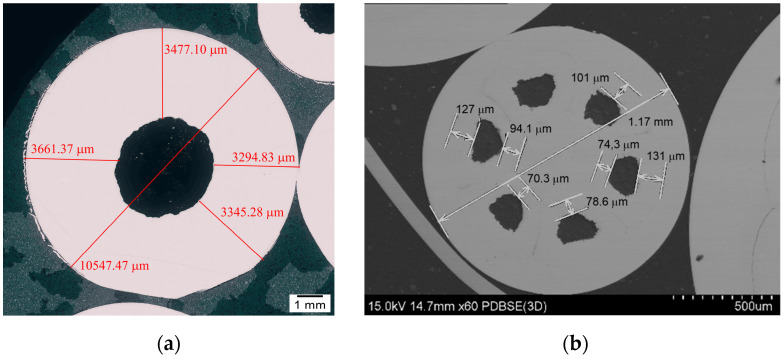
Analysis of changes in coating thickness of (**a**) single- and (**b**) multi-core wires carried out during the deformation process.

In Figure 6a, we can observe an example of the cross-section of a single-core wire with a diameter of 10.5 mm. It is evident that the core constitutes approximately 12% of the total cross-sectional area of the wire. Therefore, the predominant element of the wire area is the CuAg0.1 sheath.

**Figure 7:**
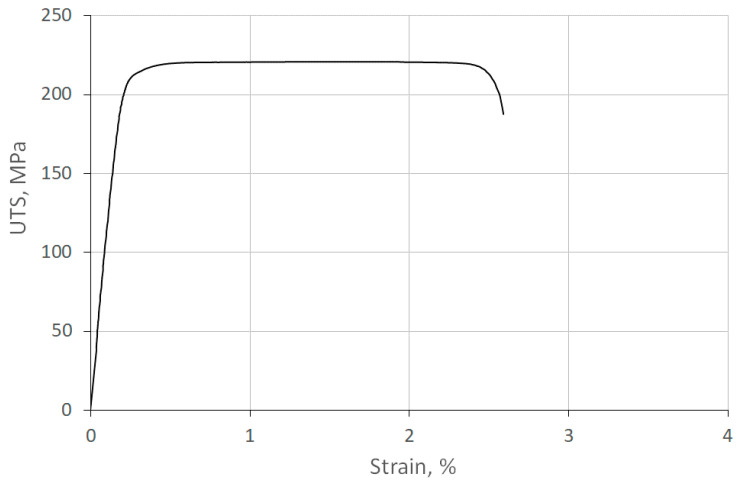
Tensile test results for sample A_Mono wire.

The results of the tensile test for a single-core wire are illustrated in Figure 7, demonstrating good strength properties and relatively high plasticity.

In the manufacturing of multicore superconducting wires, the coefficient of elongation during hydro-extrusion is approximately 4.8. During the single-core wire drawing process, this coefficient increases to about 10. However, for multicore wire drawing, the coefficient of elongation reaches approximately 110. This indicates that an initial length of around 110 mm for both the core and the sheath in a billet before hydro-extrusion results in approximately 3.8 m of single-core wire and an impressive 422 m of multicore wire. The scalability and efficiency of this process make it highly suitable for industrial applications, enabling the production of extended lengths of superconducting wires.

### 3.2. Microstructure Studies

#### 3.2.1. Microstructure After Wire Drawing Process and Before Final Heat Treatment

**Figure 8:**
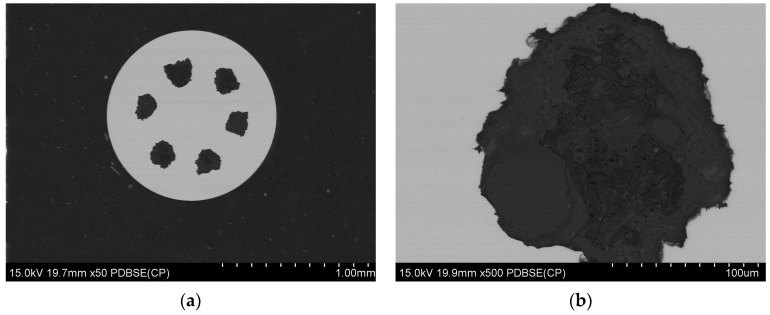
Microstructure of multi-core wire (A_Multi) with a diameter of 1.17 mm, (**a**) a cross-sectional photo of a whole multi-core wire, (**b**) zoom in on a selected single wire core.

**Figure 9:**
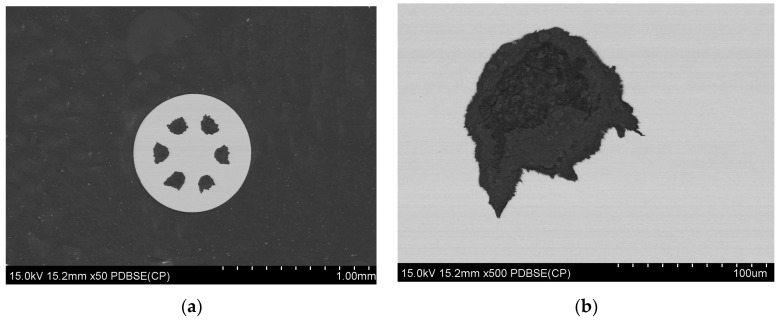
Microstructure of multi-core wire (B_Multi) with a diameter of 0.80 mm, (**a**) a cross-sectional photo of a whole multi-core wire, (**b**) zoom in on a selected single wire core.

The microstructure of the samples was prepared by cutting with a Struers Secotom cutting machine under liquid coolant, followed by polishing using a Struers Labopol polishing machine. Microstructural analysis and the examination of the chemical composition of the tested composites were conducted using a scanning electron microscope (SEM), specifically the SU-70 model from Hitachi, Tokyo, Japan, equipped with the NORAN System 7 X-ray microanalysis system (Thermo Fisher Scientific, Waltham, MA, USA) for energy-dispersive X-ray spectroscopy (EDS). The samples were prepared and tested in cross-section, and images were captured in the backscattered electrons (BSE) mode. This analytical approach facilitated the assessment of the cohesion of the coating materials and the continuity of the powder layers within the multi-core system. Refer to Figure 8 and Figure 9 for visual representations of the obtained results.

**Figure 10:**
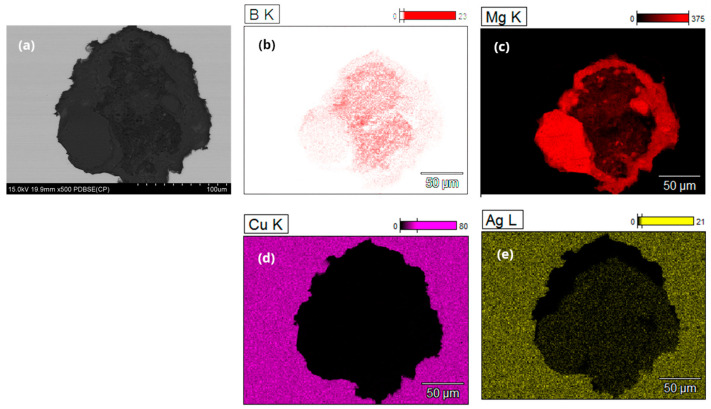
EDS microanalysis of the B, Mg, Cu, and Ag distribution in a multicore wire with a diameter of 1.17 mm (variant A_Multi), (**a**) detailed analysis of a selected core in a multi-core wire, (**b**) boron analysis of a selected core, (**c**) magnesium analysis of a selected core, (**d**) copper analysis of a selected core, (**e**) silver analysis of a selected core.

**Figure 11:**
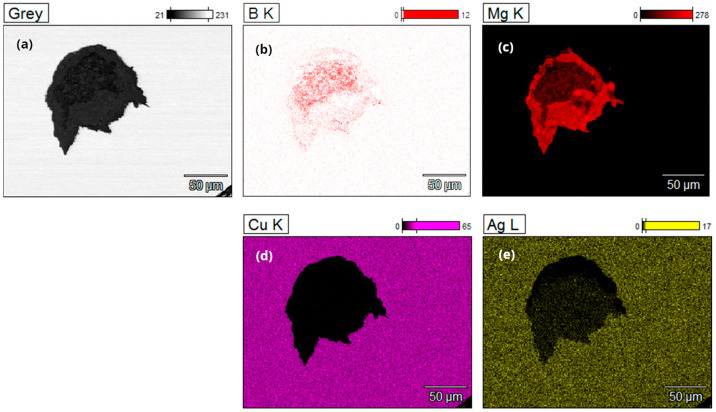
EDS microanalysis of the B, Mg, Cu, and Ag distribution in a multicore wire with a diameter of 0.80 mm (variant B_Multi), (**a**) detailed analysis of a selected core in a multi-core wire, (**b**) boron analysis of a selected core, (**c**) magnesium analysis of a selected core, (**d**) copper analysis of a selected core, (**e**) silver analysis of a selected core.

EDS microstructural analysis was conducted on cross-sectional samples of multi-core wires from both the A_Multi and B_Multi variants, revealing the distribution of the main elements in the material. These include copper and silver in the coating material, as well as boron and magnesium in the powder core (Figure 10 and Figure 11).

#### 3.2.2. Microstructure After Final Heat Treatment Process

**Figure 12:**
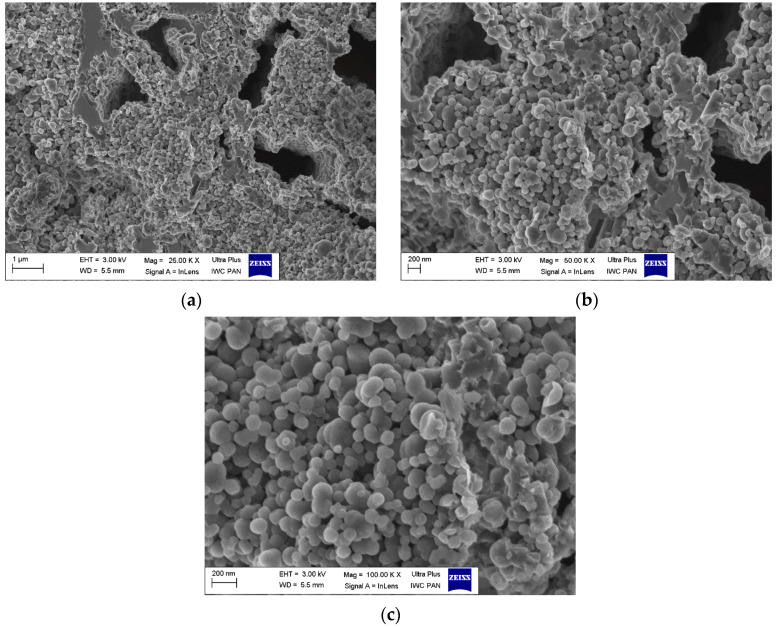
Cross-section obtained using a scanning electron microscope of sample A_Mono after annealing at *T* = 600 °C, *t* = 4 h and *p* = 0.1 MPa in a flow of argon gas. Image of a single wire core: (**a**) magnification of 25K ×; (**b**) magnification of 50K ×; (**c**) magnification of 100K ×.

**Figure 13:**
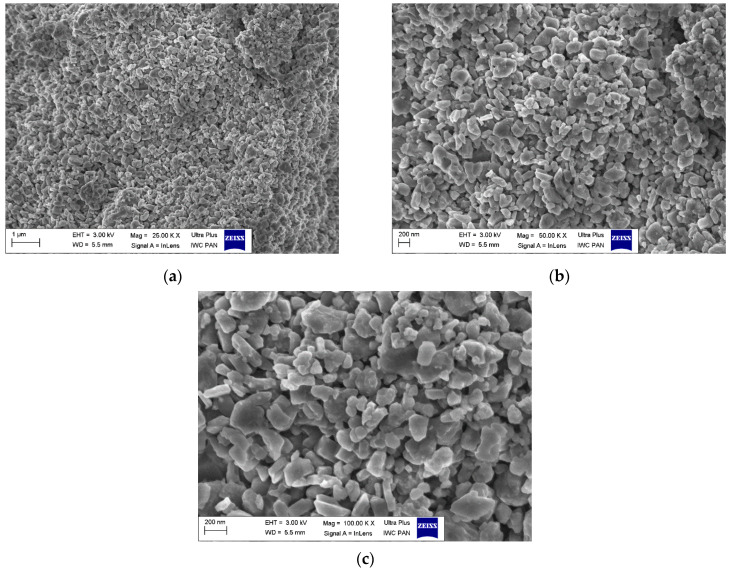
Cross-section obtained using a scanning electron microscope of sample A_Mono after annealing at *T* = 600 °C, *t* = 4 h, and *p* = 0.1 MPa in a flow of argon gas. Image of a single wire barrier: (**a**) magnification of 25K ×; (**b**) magnification of 50K ×; (**c**) magnification of 100K ×.

The microstructure analysis of the obtained wires was conducted using a Zeiss Ultra Plus scanning electron microscope. Sample photos are provided below for reference. Based on the obtained results, the microstructures of the multicore wires A_Multi and B_Multi were found to be very similar. Therefore, the results for sample A were chosen for further interpretation. Figure 12 illustrates the image of the wire core, i.e., the in situ material after heat treatment, while Figure 13 displays the image of the barrier, i.e., the ex situ material also after heat treatment. Upon scrutinizing the core using scanning electron microscopy (Figure 12a,b), numerous voids were observed in the material. The presence of such voids indicates that magnesium reacted with boron, causing magnesium contraction and the formation of a superconducting phase. In this process, grains of regular, spherical shape were generated, featuring a significant number of inter-grain connections (Figure 12c). These connections contribute to a high critical current density.

The microstructural analysis of the barrier material revealed an absence of holes or cracks (Figure 13a,b), presenting a uniform surface. This outcome is attributed to the utilization of previously reacted material. Subsequent reheating does not induce further reactions between boron and magnesium, preventing the formation of additional MgB_2_ superconducting phase. High-magnification analysis (100,000×), depicted in Figure 13c showcased large, irregularly shaped grains with sharp edges, resulting in a reduction in the number of inter-grain connections. Such a microstructure is highly desirable for a multi-core wire, as it ensures that the critical current predominantly flows through the core rather than the barrier.

### 3.3. Critical Temperature and Criticl Current Density Measurements

**Figure 14:**
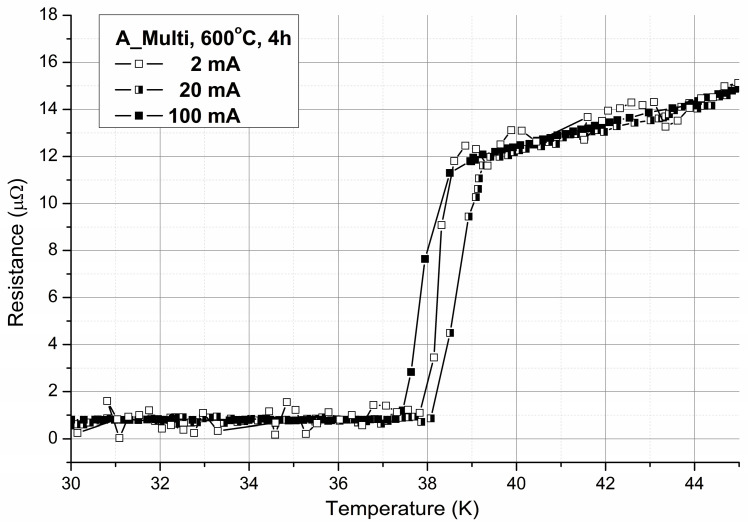
R(T) dependence at the superconducting transition for different applied currents.

**Figure 15:**
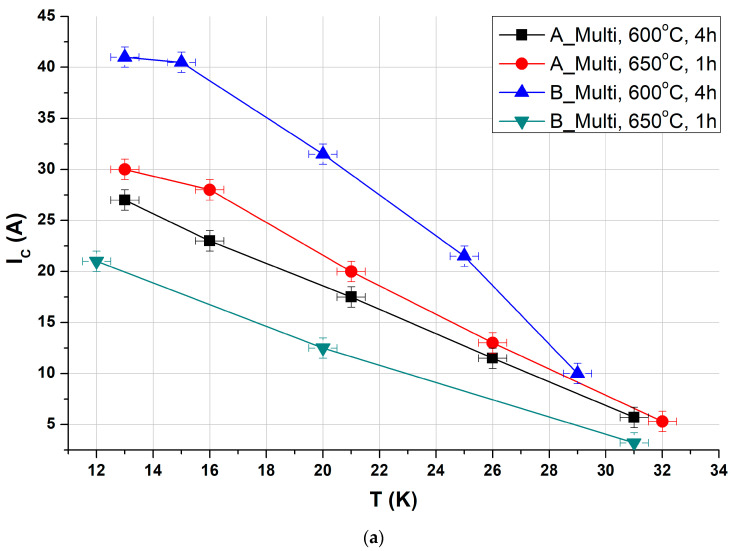

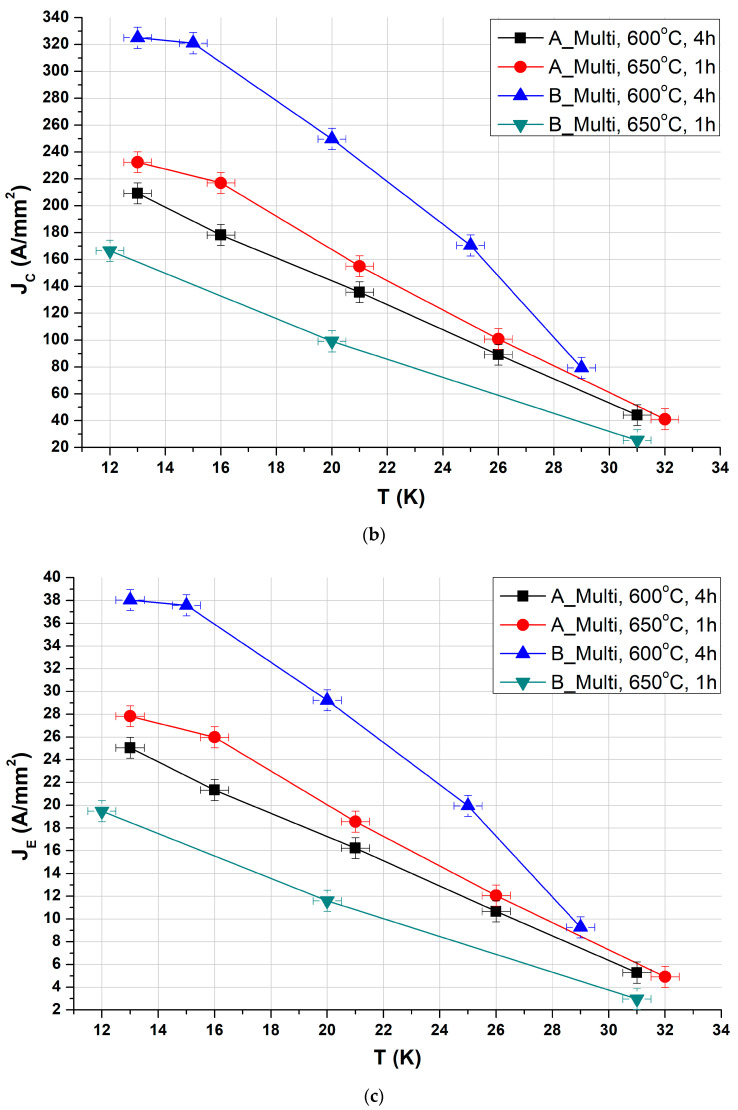
Critical current properties of wire samples measured by 4-probe transport method at various temperatures in self-field. The figures present (**a**) critical current *I_C_*, (**b**) critical current density *J_C_* in the superconductor area, and (**c**) engineering current density *J_E_* in the entire wire area.

The critical temperature (*T_c_*) was determined using the four-probe transport method with a current of 20 mA. The obtained values were normalized to the resistance at 45 K, just above the superconducting transition. Figure 14 presents examples of resistance changes as a function of temperature for different applied currents. All samples exhibited a sharp transition, indicating good electrical connectivity between grains of the superconducting material. The measured *T_c_* values were approximately 38 K, representing a favorable outcome for MgB_2_ wires. The critical current properties measured in the self-field are illustrated in Figure 15. Different samples displayed similar parameters in terms of current density. The highest values achieved at 20 K are around 250 A/mm^2^ for critical current density (*J_c_*) and 29 A/mm^2^ for engineering current density (*J_E_*). While these values are sufficiently high for self-field applications such as DC power lines, further enhancements in performance are needed to enable their use in generating a magnetic field.

## 4. Discussion

In this presented work, each stage of the process involved in producing multi-core superconducting wires with an in situ MgB_2_ core, an ex situ MgB_2_ barrier, and a CuAg0.1 sheath has been outlined.

The introduced concept of producing in situ MgB_2_ superconducting wire with an ex situ MgB_2_ barrier allows for the utilization of a copper sheath, thereby mitigating the reactivity between core materials and the sheath. The barrier not only provides protection between the materials but also exhibits superconductivity itself. The incorporation of copper renders the entire material more cost-effective compared to wires with niobium sheaths, while maintaining comparable current parameters.

For successful large-scale implementation, each production step must be economically viable and optimized. To achieve this, the hydro-extrusion process was chosen as a rapid drawing method to transform rods from 70 mm or 26 mm diameter capsules with superconducting material into 30 mm or 12 mm diameter bars, respectively.

The subsequent critical stage in wire production involved drawing the prepared bars to the final diameters of 1.17 mm and 0.8 mm. This stage serves as an indicator of the appropriateness of the selected and prepared starting materials, revealing the extent to which the superconducting material fills the wire and whether any tearing or cracking occurs. Tearing issues, commonly encountered when using a pure copper sheath, were addressed by employing a copper shield with the addition of silver, resulting in a fourfold strengthening of the shield material.

Ultimately, the multi-core wires obtained through compaction demonstrated favorable superconducting properties, including critical temperature and critical current. While these values are not yet fully optimal for contemplating industrial production of coils generating strong magnetic fields, they are already suitable for applications in DC power lines. Ongoing efforts are focused on continuous improvement to further enhance performance.

## 5. Conclusions

From the presented research, we can conclude that the process of producing multi-core superconducting wires with an in situ MgB_2_ core, an ex situ MgB_2_ barrier, and a CuAg0.1 sheath has been successfully developed. The hydro-extrusion process was effectively utilized for the rapid drawing of rods into bars. A CuAg0.1 alloy sheath was found to be an effective protective barrier between the superconducting material and the sheath, eliminating the need for a more expensive niobium sheath. The multi-core wires exhibited favorable superconducting properties, with a critical temperature (*T_c_*) of approximately 38 K, critical current density (*J_C_*) of 250 A/mm^2^ at 20 K, and an engineering current density (*J_E_*) of 29 A/mm^2^ at 20 K. Obtained values are sufficient for applications in DC power lines, but further enhancements are needed for applications in generating strong magnetic fields.

Overall, the study provides evidence of the feasibility of developing high-performance superconducting wires.

## Figures and Tables

**Table 1:** Parameters of the laboratory process of casting 26 mm diameter rods from CuAg0.1 alloy.

The Temperature of the Liquid Metal in the Crucible	The Temperature of the Bars After Leaving the Mold	Continuous Casting Speed	Mold Cooling Water Temperature	Mold Cooling Water Flow
1180–1190 °C	50–60 °C	0.3 m/min.	10–12 °C	3–3.5 L/min.

**Table 2:** Strength and electrical properties of the CuAg0.1 alloy sheath material (data for 20 °C).

Density	Brinell Hardness	Ultimate Tensile Strength	Yield Stress	Elongation to Break During Tensile Test	Electrical Conductivity	Relative Conductivity
ρ, g/cm^3^	HB	UTS, MPa	YS, MPa	A_200_, %	σ, MS/m	%IACS
8.94	85	195	120	38.5	58.0	100

**Table 3:** Basic parameters of the cold hydrostatic extrusion HE of the samples.

Notation/HE Pass	Billet Diameter *d*_0_ (mm)	Product Diameter *d_f_* (mm)	True Strain *ɛ* = ln*R* ^1^	Extrusion Pressure *p_HE_* (MPa)	Measurement Error (Standard Deviation)
A_Mono	26.02	11.91	1.56	576	±16.68
B_Mono					

^1^ *R*—reduction ratio = initial to final cross section.

**Table 4:** Schematic table showing the deformation of single- and multi-core wires during the drawing process, from an initial diameter of 12.15 mm to a final diameter of 1.17 mm (diameter values in mm).

12.15	11.55	11.02	10.50	10.00	9.50	9.10	8.70	8.35	8.00	7.65	7.30	6.97
6.65	6.32	6.05	5.77	5.50	5.30	5.00	4.77	4.55	4.35	4.15	3.96	3.78
3.61	3.44	3.29	3.14	3.00	2.86	2.73	2.61	2.49	2.37	2.26	2.16	2.07
1.97	1.87	1.78	1.70	1.62	1.55	1.49	1.42	1.36	1.29	1.23	1.17	

**Table 5:** Parameters of process of mono- and multi-core wire drawing.

Sample	Cone Angle 2α	Single Operation Elongation	Wire Drawing Speed	Diameter Range	Total True Strain
	°	%	m/s	mm	-
A_Mono B_Mono A_Multi B_Multi	14	10	0.2	12.15–1.17	4.7

**Table 6:** Lubrication during the deformation process of single-core and multi-core wires.

Sample	Type of Material	Lubricant
A_Multi	Multicore, 6 + 1, without heat treatment	Multidraw Cu MF (Zeller + Gmalin) oil: density 950 kg/m^3^, viscosity 75 mm^2^/s, emulsion: pH 9.0, electrical conductivity 1100 µS/cm
B_Multi	Multicore, 6 + 1, with heat treatment: 350 °C/1 h	

**Table 7:** Obtained MgB_2_ samples after drawing process and final heat treatment for in situ MgB_2_ synthesis.

Sample	Type of Wire	Diameter [mm]	Heat Treatment Process
A_Multi_600 °C_4 h	7-core with Cu outer coating, including: 6 cores—in situ with ex situ barrier and CuAg0.1 sheath; center core—Cu	1.17/0.80	*T* = 600 °C; *p* = 0.1 MPa; *t* = 4 h
B_Multi_600 °C_4 h			
A_Multi_650 °C_1 h	7-core with Cu outer coating, including: 6 cores—in situ with ex situ barrier and CuAg0.1 sheath; center core—Cu	1.17/0.80	*T* = 650 °C; *p* = 0.1 MPa; *t* = 1 h
B_Multi_650 °C_1 h			

**Table 8:** Hardness test results.

	The Average HV5 Hardness Value of the Outer Coating and Sheath Material (kgf/mm^2^)							
Wire Diameter	12.15 mm		4.15 mm		3 mm		1.17 mm	
Outer Coating/Core Sheath Material	Cu ETP	CuAg0.1	Cu ETP	CuAg0.1	Cu ETP	CuAg0.1	Cu ETP	CuAg0.1
A_Multi	108.1	111.2	120.4	125.1	122.3	127.5	129.4	136.3
B_Multi	113.3	115.6	123.0	129.5	128.7	134.1	134.8	141.1

## Data Availability

The original contributions presented in this study are included in the article. Further inquiries can be directed to the corresponding authors.
